# Intrapancreatic Accessory Spleen Masquerading as a Pancreatic Mucinous Neoplasm

**DOI:** 10.1055/s-0040-1710342

**Published:** 2020-06-16

**Authors:** Shiva Poola, Shachar Laks, Peter Kragel, Kara Regan

**Affiliations:** 1 Department of Internal Medicine/Pediatrics, Vidant Medical Center/Brody School of Medicine, Greenville, North Carolina; 2 Deparment of Surgery, Vidant Medical Center/Brody School of Medicine, Greenville, North Carolina; 3 Deparment of Pathology, Vidant Medical Center/Brody School of Medicine, Greenville, North Carolina; 4 Deparment of Gastroenterology, Vidant Medical Center/Brody School of Medicine, Greenville, North Carolina

**Keywords:** pancreatic neoplasm, intrapancreatic accessory spleen, endoscopic ultrasound

## Abstract

Incidentally discovered pancreatic cysts have become more common with increasing use of abdominal cross-sectional imaging. Tools that help us to better risk stratify a pancreatic cyst include advanced imaging techniques, such as pancreatic protocol computed tomography (CT) scan or magnetic resonance imaging (MRI) with cholangiopancreatography. Endoscopic ultrasound (EUS) and fine-needle aspiration (FNA) are invasive measures to better define and sample cysts especially if high-risk features are present. EUS may also yield pancreatic cyst fluid for analysis of carcinoembryonic antigen (CEA) which is elevated in mucinous cysts. This case highlights a rare finding of a mucinous, epidermoid cyst in an intrapancreatic accessory spleen (IPAS) with high-risk features on EUS.

Incidentally discovered pancreatic cysts have become more common with increasing use of abdominal cross-sectional imaging. Many of these cysts are benign, while some are malignant or have malignant potential. Further evaluation and management of asymptomatic pancreatic cysts depends on its malignant potential. Tools that help us to better risk stratify a pancreatic cyst include advanced imaging techniques such as pancreatic protocol computed tomography (CT) scan or magnetic resonance imaging (MRI) with cholangiopancreatography. Endoscopic ultrasound (EUS) and fine-needle aspiration (FNA) are invasive measures to better define and sample cysts especially if high-risk features are present. High-risk features within a cyst, such as nodularity, calcifications, or pancreatic ductal dilatation, are concerning. These may suggest advanced dysplasia or malignancy. These lesions are strongly considered for surgical resection due to malignant potential even when needle aspiration does not demonstrate concerning findings due to the low sensitivity. 1 EUS may also yield pancreatic cyst fluid for analysis of carcinoembryonic antigen (CEA) which is elevated in mucinous cysts. This case highlights a rare finding of a mucinous, epidermoid cyst in an intrapancreatic accessory spleen (IPAS) with high-risk features on EUS.

## Case Presentation

**Fig. 1:**
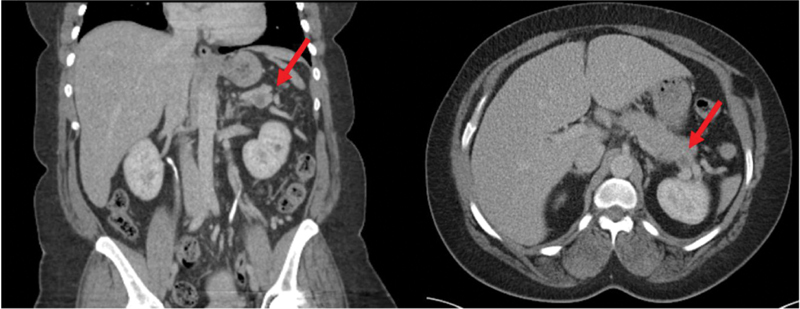
Pancreatic lesion (arrow) on computed tomography imaging.

**Fig. 2:**
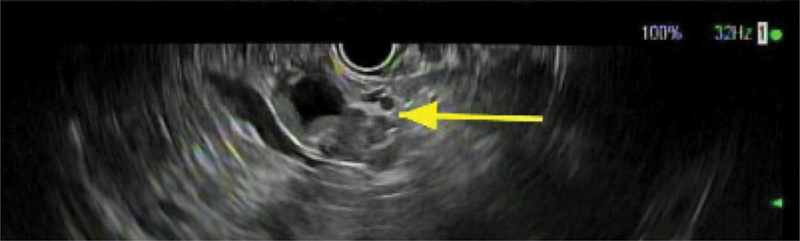
Atypical cystic pancreatic tail lesion on EUS with solid components. EUS, endoscopic ultrasound.

This is a 42-year-old African American female who had an incidental finding of a new 17-mm pancreatic tail cystic lesion found on a CT scan during workup for abdominal pain. Patient was lost to follow-up, until a repeat CT scan 1 year later demonstrated a stable low attenuation, 15 mm × 11 mm pancreatic tail cyst ( Fig. 1 ). She complained of decreased appetite and intermittent epigastric pain over the last year. She was referred for EUS with FNA for further evaluation of the cyst. Her history is significant for diabetes mellitus, human immunodeficiency (well-controlled), alcohol and intermittent substance usage (marijuana and cocaine), recent ventral hernia repair, and family history significant only for breast and lung cancer. She has no significant tobacco usage history. No prior episodes of pancreatitis, known pancreatic disorder, or prior intervention. Exam is otherwise unremarkable, without palpable mass, jaundice, or tenderness to palpation. The patient underwent an EUS which found a complex cystic lesion in the pancreatic tail and an abnormal lymph node in the peripancreatic region ( Fig. 2 ). The pancreatic lesion had high-risk features, demonstrating both cystic and atypical solid components, measuring 27 mm × 9 mm, and abutting the splenic vessels near the hilum without invasion and a 27 mm × 11 mm enlarged peripancreatic lymph node. Both were sampled by FNA. Approximately 5 mL of pancreatic cystic fluid was obtained which appeared cloudy, blood tinged, and viscous. Fluid analysis demonstrated CEA level of 5,327.7 ng/mL, amylase of 335 U/L, and glucose of 69 mg/dL. Cytology demonstrated benign appearing squamous cells and a few atypical, degenerated cells. Lymph node findings were benign. Serum CA 19–9 was low at <3 U/L.

**Fig. 3:**
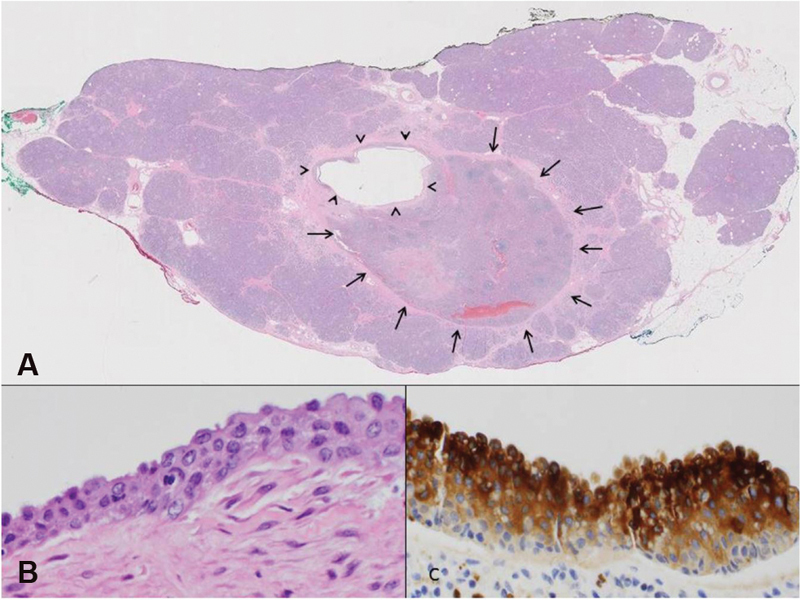
Pathology of intrapancreatic cyst. ( **A** ) Microscopic examination of the distal pancreas shows and intrapancreatic accessory spleen (arrows) and an associated epithelial cyst (arrowheads, hematoxylin and eosin, original magnification ×2). ( **B** ) Higher power shows a stratified epithelial lining without keratinization. Some cells abutting the cystic space demonstrate apical nuclei while in other cells there appear to be cytoplasmic blebs (hematoxylin and eosin, original magnification ×400). ( **C** ) The linking epithelial is strongly positive on immunostaining for polyclonal carcinoembryonic antigen. (immunohistochemistry with DAB chromogen, original magnification ×400). DAB, 3,3′-Diaminobenzidine.

Given these high-risk findings the patient underwent distal pancreatectomy and splenectomy. Intraoperative findings included no evidence of distant metastatic disease, no worrisome lymphadenopathy, and a lesion in the pancreatic tail without invasions into surrounding tissues. Pathological evaluation demonstrated IPAS with associated benign epithelial-lined mucinous cyst, without in situ or invasive carcinoma identified. The cyst lining shows squamoid and apocrine features. No goblet cells are identified. Immunohistochemistry shows positive staining for CEA in the cyst lining ( Fig. 3 ). Twelve benign lymph nodes negative for carcinoma and negative margins. The patient did well postoperatively and was discharged on postoperative day 6. At outpatient postoperative visit, she continued to do well and had no ongoing issues with abdominal pain.

## Discussion

IPAS is found in approximately 10% of autopsies. Accessory spleens are due to postoperative splenosis or ectopic proliferation of splenic tissue during fetal development. 2 IPAS may frequently be detected as a nodule on CT and MRI imaging characterized similar to the spleen on precontrast and contrast-enhanced imaging modalities. 3 Similarly on ultrasound imaging, accessory spleens are echogenically similar to the main spleen and are round or oval with presence of vascular hilum on Doppler ultrasound. 4 Other studies have suggested the usage of contrast-enhanced EUS and EUS-elastography as a useful tool for diagnosis of IPAS. 5 Appearance of the lesion in this case was a very unusual presentation for anIPAS, given its association within a mucinous cyst. This is a rare diagnosis with less than 60 reported cases. Malignant potential is unknown; however, there is a report of squamous cell carcinoma arising in an epithelioid cyst within the spleen. 6

Epithelial cysts in IPAS is rare with half of these reported incidentally detected. Zavras et al reviewed 36 patients and found the mean age of patients to be 46 years and slightly over half were female. Patients were asymptomatic or complained of abdominal or epigastric pain. The cysts were all located in the tail of the pancreas and ranged in size from 1.4 to 12.6 cm. Serum CEA and CA 19–9 levels were normal in the majority of patient and immunohistochemistry demonstrated positivity for CEA in the cyst lining. 7 Similar findings were found in this case. Her serum CEA was normal and immunohistochemistry was positive staining for CEA. Epithelial cysts of the spleen demonstrate an epithelial lining of low cuboidal, low columnar, or squamous type, surrounded by splenic tissue. 8 The cyst lining of epithelial cysts in IPAS shows similar histology which was also found in our patient.

Fluid analysis and serum biomarkers are widely used for the evaluation of pancreatic masses. However, the efficacy of fluid studies continues to be poorly demonstrated in IPAS with less than 20% of cases reporting fluid biomarkers. 9 10 Li et al conduced a systematic review of 56 patients with epidermoid cysts in IPAS and found 9 of 9 patients with an elevated cyst fluid CEA and 1 of 6 patients with an elevated fluid CA 19–9. Serum CEA was normal in 26 patients compared with only one patient with an elevated serum CEA. Reported serum CA 19–9 levels were elevated in 20 of 37 patients. 11 Despite these findings, there is insufficient data to conclude significance of these biomarkers in epidermoid cystic IPAS.

Appropriate management of mucinous epidermoid cyst in IPAS has not been established. A study in Spain found four cases of IPAS that were diagnosed with EUS and FNA which were safety followed with imaging studies. 12 However, all reported cases of epidermal cysts in IPAS have undergone surgical resection or excision. 13 Conservative management of these lesions has yet to be reported or studied.

## Conclusion

In conclusion, IPAS within a mucinous epithelioid cyst is a rare entity in the literature. Imaging and endoscopic evaluation may be helpful in the diagnosis; however, there continues to be little information of appropriate management. Specific biomarkers have yet to identify to separate IPAS from other malignant potential mucinous cysts of the pancreas. Future case series regarding radiographic imaging, cystic features, and specific fluid biomarkers may be required to fully understand Epidermoid Cyst in IPAS and identify malignant potential.
